# What is the health status of girls and boys in the COVID-19 pandemic? Selected results of the KIDA study

**DOI:** 10.25646/11436

**Published:** 2023-06-14

**Authors:** Julika Loss, Miriam Blume, Laura Neuperdt, Nadine Flerlage, Tim Weihrauch, Kristin Manz, Roma Thamm, Christina Poethko-Müller, Elvira Mauz, Petra Rattay, Jennifer Allen, Mira Tschorn

**Affiliations:** 1 Robert Koch Institute, Berlin Department of Epidemiology and Health Monitoring; 2 University of Potsdam, Social and Preventive Medicine, Department of Sports and Health Sciences

**Keywords:** CHILD AND ADOLESCENT HEALTH, GENDER, COVID-19 PANDEMIC, PHYSICAL ACTIVITY, MENTAL HEALTH

## Abstract

**Background:**

It is well known that there are gender differences in the health behaviour and physical and mental health of children. The COVID-19 pandemic influenced the health and lifestyles of children and adolescents by changing their living conditions. The present work investigates whether gender differences in selected health indicators are evident more than two years after the onset of the pandemic.

**Methods:**

In the study Kindergesundheit in Deutschland aktuell (KIDA) (German Children’s Health Update), cross-sectional telephone surveys were conducted with parents of 3- to 15-year-olds (n=3,478). Parental information on the general and mental health of the child, on increased need for health care and mental health services, as well as on physical activity and utilisation of sports activities were queried in standardised manner. Gender differences were assessed using Chi^2^ tests.

**Results:**

A total of 91% of the girls and 92% of the boys had their general health assessed as being (very) good by their parents (difference not significant, n.s.). An increased need for care and support was indicated for 10.6% of the 3- to 15-year-olds (girls: 9%, boys: 12%, n.s.). Boys met the physical activity recommendations of the WHO significantly more often (60%) than girls (54%). Good to excellent mental health was reported for 93% of both boys and girls. When changes during the pandemic were reported, no differences were found in the responses for girls compared to boys.

**Conclusions:**

Gender differences were found for individual parameters and age groups. These differences must be assessed in the context of other social determinants of health, and need to be considered when planning preventive measures.

## 1. Introduction

In late 2019, the novel coronavirus (SARS-CoV-2) was identified, resulting in a multi-year pandemic. To reduce the risk of disease by contracting SARS-CoV-2, governments around the world implemented varying containment measures, ranging from recommendations to wear a face mask, social distancing measures, to broader lockdowns as well as school and daycare closures, depending on the phase of the pandemic [1]. In the course of the COVID-19 pandemic, these measures repeatedly led to a variety of changes in the living conditions of children and adolescents, such as short-term closures of schools and day-care centres, restrictions on contact with classmates or friends, as well as on leisure activities and social activities. As such, these mitigation measures also influenced the physical and mental health and health behaviour of children and adolescents [2–9]. Delivery of medical care was also repeatedly limited, for the purpose of infection control or due to capacity bottlenecks caused by the pandemic. Further negative factors influencing health could have been caused by family distress, such as when jobs were lost in context of the COVID-19 pandemic, with low-income parents more likely to be affected [10–12]. Long-term effects on children’s health may also result if children or family members experience severe SARS-CoV-2 infection of and if symptoms persist for a long time (so-called post-COVID syndrome) [13, 14].

We know from studies conducted before the COVID-19 pandemic that there are gender-related differences in the health behaviour and physical and mental health of children and adolescents. For example, the KiGGS study showed that girls are generally healthier in childhood – both in terms of parent-reported general health and disease frequencies – and exhibit healthier behaviour than boys. In adolescence, on the other hand, the physical and mental health status of female adolescents tends to be worse than that of their male counterparts, as they experience stress more often and engage less in sports activities. However, they have a healthier diet than male adolescents [15–18]. These gender differences are due not so much to biological factors, but rather to different living conditions of boys and girls, whereby gender-related socialisation, gender roles and images and thus associated expectations of femininity and masculinity are also of crucial importance [17]. Gender-related differences are also evident in leisure activities and social interactions [19, 20]. As some of these areas were significantly affected by the COVID-19 pandemic, differences in the effects on girls and boys can be expected. Previous studies also point to age- and gender-related differences in the response to stressors [21]. Against this background, it is important to investigate whether gender differences exist with regard to various health outcomes in the current pandemic scenario. For example, results of a study from Germany on mental health show that a greater proportion of girls had a reduced health-related quality of life during the COVID-19 pandemic as compared to boys [3, 4]. Several studies on physical activity of children have shown that boys reduced their physical activity more than girls during the pandemic [22, 23]. This may be due, for example, to the fact that boys are more likely to participate in organised team sports, which were temporarily suspended during the pandemic.

The fact that everyday life is largely shaped by the infection scenario and, at times, by containment measures was considered a temporary exceptional situation at the onset of the pandemic. However, as the COVID-19 pandemic continued over several years, this situation has become the ‘new normal’. Many children have now spent a large part of their lives in the pandemic. Some effects caused by containment measures may be receding; for example, it is conceivable that children’s physical activity behaviour may return to normal as daycare centres, sports fields and sports clubs reopen. Other health changes, such as an increase in anxiety symptoms or an increased need for medical care, may have become more permanent. It therefore seems sensible to survey the health status of children and adolescents as a status quo after approximately three years of the pandemic and to continue monitoring it in the future, including in a post-pandemic phase.

The investigation of age- and gender-related differences in health status during the COVID-19 pandemic is important in order to be able to develop effective interventions for now and also in the future that are adapted to the special circumstances of boys and girls in various age groups in epidemically significant social scenarios or crises [24–26]. Health promotion and prevention should not be ‘gender-blind’ in childhood and adolescence, but rather should take into account for the possibility of boys and girls having special needs. This can be implemented in various ways, for example by approaching adolescent boys and girls differently in educational campaigns [27], or through gender-homogeneous offers in areas such as sports or healthy nutrition [28]. In environmental and structural approaches, e.g. in school settings, attention should also be paid to whether or not both genders benefit equally [29]. If, for example, new sports and play areas in the school’s playground are mainly used by (athletic) boys, and girls are not given the opportunity to be active there, gender differences are increased rather than diminished [29]. Health promotion measures should therefore be targeted to reduce gender-related health inequalities. For this purpose, it is important to recognise existing inequalities.

Therefore, data from the Kindergesundheit in Deutschland aktuell (KIDA) (German Children's Health Update) study, which was collected from February – October 2022 from parents of children and adolescents between 3 and 15 years of age, were analysed with regard to gender-related differences. The following questions are in focus of this article:

Are there differences between boys and girls in terms of

► general health and subjective mental health?
► need for care and support?
► physical activity and utilisation of physical activity programmes?

In part, we will also consider whether those gender-related differences that were already described for children and adolescents in pre-pandemic times continue to exist, and whether changes observed during the COVID-19 pandemic concern girls and boys to the same extent.

**KIDA**

Survey on children’s health in Germany (German Children's Health Update)

**Data holder:** Robert Koch Institute

**Objectives:** provision of reliable information on the physical and mental health status and health behaviors of children and adolescents aged 3 to 17 years.

**Study design:** nationwide cross-sectional telephone survey and follow-up in-depth online survey

**Population:** parents of children aged 3–15 years and adolescents aged 16–17 years who are integrated in the ongoing GEDA study.

**Sampling:** random sample of landline and mobile phone numbers (dual frame method) from the sampling system of the ADM (Arbeitskreis Deutscher Markt- und Sozialforschungsinstitute e.V.)

**Sample size:** approx. 7,000 participants

**Study period:** February 2022 – June 2023

Further information in German is available at www.rki.de/kida

## 2. Methods

### 2.1 Study design and study procedure

Within the framework of the Kindergesundheit in Deutschland aktuell (KIDA) (German Children's Health Update) study, continuous (cross-sectional) telephone surveys were conducted from February to October 2022. The part of the populace living in private households with children and adolescents between 3 and 15 years of age who stated Germany as their current usual place of residence served as the base population of the study. In each case, one parent was interviewed about the health of the children, i.e. all information about the health situation of the children and adolescents was collected on a parent-reported basis. KIDA was embedded in the Gesundheit in Deutschland aktuell (GEDA 2022) study [30]. GEDA is a telephone survey of the adult populace living in Germany that is representative of the population and has been conducted regularly since 2008. For the sampling, the telephone sampling system of the Arbeitskreis Deutscher Markt- und Sozialforschungsinstitute e.V. (ADM) is used based on two selected populations: a mobile phone population and a fixed network population. Based on this access, all persons with children between 3 and 15 years of age living in the household as well as adolescents aged 16 years and older are included for participation in the telephone survey of the KIDA study.

The survey was conducted using a standardised questionnaire. The responses of the participants were recorded in computer-aided manner by trained interviewers using the ‘VOXCO Interviewsuite’ software. Data for up to two children or adolescents living in the household were obtained for KIDA. For households with more than two children, the selection of the children to report on was random. Information on two children or adolescents from the same family was taken into account in the design and adjustment weighting via a household variable.

### 2.2 Indicators

For recording of the internationally established indicator of general health, parents were asked in accordance with a recommendation of the World Health Organisation (WHO) [31]: ‘How would you describe your child‘s state of health in general?’ [32] The parents responded using a five-point scale, which was combined into the categories of ‘very good/good’ and ‘average/bad/very bad’ for the present evaluations.

Parent-reported subjective mental health was measured by an established single item [33]: ‘How would you rate your child‘s mental health in general?’ The response options were: ‘excellent’, ‘very good’, ‘good’, ‘fair’, ‘poor’. Analogous to the surveillance systems in Canada [34] and Australia [35], the two categories ‘excellent’ and ‘very good’ were combined. In addition, a differentiation was made between ‘good’ and ‘fair’ or ‘poor’ mental health. Furthermore, parents were queried about the change in their children‘s mental health compared to the time before the onset of the COVID-19 pandemic in Germany: ‘Compared to the time before the Corona pandemic (i.e. before March 2020), how would you describe your child‘s current mental health?’ The response options were: ‘much better’, ‘somewhat better’, ‘about the same’, ‘somewhat worse’, ‘much worse’. The response categories ‘much better’ and ‘somewhat better’ as well as ‘somewhat worse’ and ‘much worse’ were combined, whereas the response category ‘about the same’ was kept as ‘unchanged’ (‘improved’, ‘unchanged’, ‘worsened’).

An item from the validated German translation of the Children with Special Health Care Needs (CSHCN) screener [36, 37] was used to record an increased need for care or support. The question was: ‘Does your child need or use more medical care, mental health or educational services than is usual for most children of the same age?’ If this question was answered in the affirmative, it was followed by the questions ‘Is this because of any medical, behavioral or other health condition?’ and ‘Is this a condition that has lasted or is expected to last for at least 12 months?’. If all three questions were answered in the affirmative, an increased need for care or support was seen to be evident.

Physical activity was recorded through the question: ‘How much lively exercise has your child had in the last week?’ With response categories being ‘less than 1 hour’, ‘1–2 hours’, ‘3–4 hours’, ‘5–6 hours’ and ‘at least 7 hours’. The question is based on the WHO recommendation according to which children and adolescents from 5 to 17 years of age should be active with moderate to vigorous intensity for an average of at least 60 minutes a day [38]. In addition, it was asked whether the child had participated (a) in voluntary physical activity or sport courses at school, and (b) in sports courses at sport clubs, fitness studios, ballet or swimming schools in the previous four weeks. If the response to the question was negative, it was asked in each case why the child/adolescent did not participate in such activities with the response categories being ‘Because the sports activity programmes at school (or sports courses in sports clubs/gyms) do not take place due to the pandemic’, ‘Because the child does not participate/is not registered due to the pandemic’, and ‘Because he/she does not participate/is not registered for other reasons’. In the case of an affirmative response, it was asked whether the pandemic had changed how often the child participates in the corresponding physical activity programmes (‘less frequently’/‘more frequently’/‘no change’).

The gender of the children and adolescents was recorded through the question: ‘Which gender was entered on your child‘s birth certificate?’ The possible responses were ‘male’, ‘female’, ‘The gender entry was not completed’ or ‘diverse’. Due to missing or low numbers of cases in the categories ‘The gender entry was not completed’ and ‘diverse’, these children and adolescents are excluded when considering the genders separately. The parents‘ information on the age of their children was combined into the age groups of 3–6 years, 7–10 years, and 11–15 years.

In order to be able to generate meaningful results for Germany, a weighting factor was created for the present analyses. The weighting consists of a design weight and an adjustment weight. The design weight is determined by the selection probability of the participating person. The adjustment weighting counteracts the fact that persons from certain population groups with a lower willingness to participate are under-represented compared to the base population of the study. The weighting adjusts the sample as a whole to the population distribution of variables such as region, age, gender, and education level (CASMIN classification) [39]. For example, participants from the low education group are weighted up, giving more weight to responses from these individuals and compensating for the aspect of lower participation rates from this group.

Stratified, weighted prevalences and 95% confidence intervals are reported below for the total group and by age group. Pearson‘s Chi-square tests (Chi^2^ test) were used to assess statistical health differences between boys and girls. A p-value of <0.05 was considered statistically significant. However, the p-values were not adjusted for multiple testing, so that statistically significant group differences determined herein are to be seen as statistical anomalies of an explorative and descriptive testing rather than as confirmation of tests of hypotheses.

## 3. Results

During the survey period from February – October 2022, parent-reported data were recorded from a total of 3,478 children and adolescents between 3 and 15 years of age (girls: n=1,639, boys: n=1,838, no information provided: n=1; Table 1). For the age group of 3–6 years, responses were available for 917 children. For the age groups of 7–10 years and 11–15 years, parents provided information for 1,027 children and 1,534 children, respectively.

### 3.1 General health

**Figure 1:**
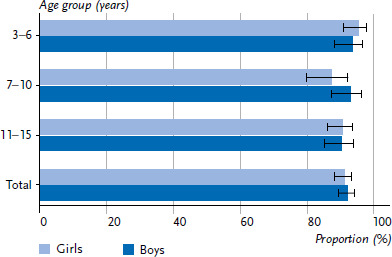
Frequency of parent-reported very good or good general health of 3- to 15-year-olds by gender and age group (total: N=3,478; girls: n=1,639; boys: n=1,838; 3–6 years: n=916; 7–10 years: n=1,027; 11–15 years: n=1,534), Proportions in percent and 95% confidence intervals Source: KIDA study (survey period 02/2022–10/2022)

A total of 92.0% of the participating parents rated the general health of their child (aged 3–15 years) as very good or good; 8.0% of the parents rated the general health of their child as moderate or poor. There was no statistically significant difference between girls and boys (p=0.63). The age-specific analysis also showed no statistically significant gender differences in the general health for the three age groups of 3–6 years (p=0.51), 7–10 years (p=0.14), and 11–15 years (p=0.98). However, it was striking that among the 7-to 10-year-olds, just less than 13% of the girls were in mediocre to poor general health, but only just less than 7% of the boys (Figure 1).

### 3.2 Subjective mental health

**Figure 2:**
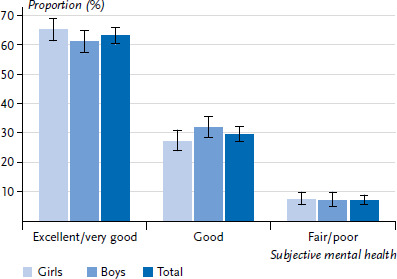
Parent-reported subjective mental health of 3- to 15-year-olds by gender (total: N=3,475; girls: n=1,638; boys: n=1,836), Proportions in percent and 95% confidence intervals Source: KIDA study (survey period 02/2022–10/2022)

Subjective mental health was rated positively by parents for a majority of children and adolescents between 3 and 15 years of age: 63.2% of parents rated their child’s mental health as ‘excellent’ or ‘very good’, and another third (29.6%) rated the mental health as ‘good’. For 7.2% of the children and adolescents, their parents rated their mental health as ‘fair’ or ‘poor’. The evaluation stratified by gender (Figure 2) showed no statistically significant differences between girls and boys (p=0.48).

When examining age-specific gender differences in subjective mental health, there were no statistically significant differences between the children and adolescents in the analysed age groups. This was true for the age group of 3-to 6-year-olds (excellent or very good: girls 79.7%, boys 74.3%, p=0.77) as well as for the age group of 7- to 10-year-olds (girls 61.5%, boys 55.9%, p=0.52) and the 11- to 15-year-olds (girls 56.0%, boys 54.4%, p=0.30).

**Figure 3:**
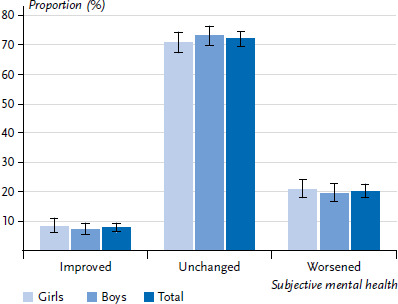
Change in parent-reported subjective mental health of 3- to 15-year-olds as compared to pre-pandemic period by gender (total: N=3,453; girls: n=1,631; boys: n=1,821), Proportions in percent and 95% confidence intervals Source: KIDA study (survey period 02/2022–10/2022)

According to parents’ assessment, the mental health of 72.0% of children and adolescents between 3 and 15 years of age remained steady during the COVID-19 pandemic as compared to the pre-pandemic period (Figure 3). Parents reported an improvement in mental health for 7.8% of children and adolescents and a deterioration for 20.2%. An evaluation stratified by girls and boys showed no statistically significant gender differences (p=0,86). Likewise, an analysis of age-stratified gender differences also showed no statistically significant differences: diminished mental health was reported for 9.9% of girls and 10.1% of boys in the age group of 3- to 6-year-olds (p=0.79), for 20.3% of girls and 20.9% of boys in the age group of 7- to 10-year-olds (p=0.96), and for 30.5% of girls and 26.3% of boys in the age group between 11 and 15 years of age (p=0.60).

### 3.3 Increased care or support needs

**Figure 4:**
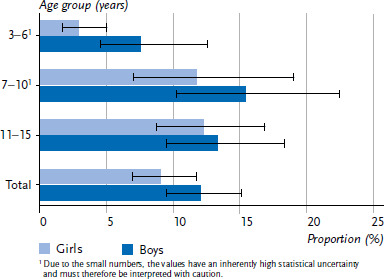
Frequency of increased care and support needs of 3- to 15-year-olds by gender and age group (total: N=3,447; girls: n=1,626; boys: n=1,820; 3–6 years: n=913; 7–10 years: n=1,013; 11–15 years: n=1,520), Proportions in percent and 95% confidence intervals Source: KIDA study (survey period 02/2022–10/2022)

Participating parents reported increased medical, mental health or educational care or support needs for 10.6% of the children in the age group of 3 to 15 years of age (Figure 4). Across all age groups, the more frequent need for care or support reported for boys (12.1%) as compared to girls (9.1%) was not statistically significant (p=0.12). When examining age-specific gender differences, a statistically significant difference was only found for children between 3 and 6 years of age (p=0.01), but there was no difference in the age groups of 7–10 years (p=0.41) and 11–15 years (p=0.73).

### 3.4 Physical activity

**Figure 5:**
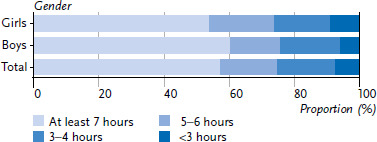
Extent of moderate to vigorous physical activity of 3- to 15-year-olds per week (referring to the week before the survey, by gender (total: N=3,456, girls: n=1,629, boys: n=1,827)) Source: KIDA study (survey period 02/2022–10/2022)

According to the responses by the parents, between January and October 2022, a total of 57% of the children and adolescents engaged in at least seven hours of moderate to vigorous physical activity per week; they thus met the WHO’s physical activity recommendation of an average of at least 60 minutes of moderate to vigorous physical activity per day (Figure 5). The proportion was statistically significantly higher for boys than for girls (60.0% versus 53.8%, p=0.02).

The gender difference in the extent of physical activity was more pronounced for younger children than for older children and adolescents. In the age group of 3–10 years, 70.2% of the boys but only 61.6% of the girls met the physical activity recommendations (p=0.01). These proportions balanced out at an older age (boys: 43.3%, girls 40.9%, no significant difference). Overall, the proportion of children and adolescents who were sufficiently active decreased with age: the WHO physical activity recommendation was met by 66.1% of the 3- to 10-year-olds and 42.2% of the 11-to 15-year-olds.

Half of the children and adolescents (54.8%, n=1,897) were active in club or commercial sports programmes in the four weeks prior to the survey; the proportion was higher for boys than for girls (57.7% versus 51.6%, p=0.04). A similar picture emerged with regard to participation in sports activity programmes (AGs) at school: a total of 52.7% of the pupils (n=1,134/2,151) took part in sports activity programmes in the four weeks before the survey: 56.5% of the boys and 48.7% of the girls (p=0.04). A total of 22.3% of the pupils did not take part in any organized sport offer, either in a sports activity programme at school or in sports clubs or studios; this was more frequent for female pupils (27.6%) than male pupils (17.2%; p<0.01).

Looking at the group of children and adolescents who (according to their parents) had been active in sports in a sports club and/or sports or dance studio in the previous four weeks (n=1,866), 22.4% of them stated that they had used the corresponding course less often due to the pandemic. There were no statistically significant gender differences in these proportions (boys 20.1% versus girls 25.1%, p>0.05). For the group of children and adolescents who had not used this kind of offer in the previous four weeks (n=1,179), 14.6% of the parents reported that this was due to the offer being cancelled due to the pandemic. There was also no difference between boys and girls (boys 14.9% versus girls 14.3%, p=0.33).

## 4. Discussion

### 4.1 Summary of the most important results

The KIDA study provides parents’ responses on the subjective general health and mental health, on the care and support needs, and on the physical activity behaviour of almost 3,500 children and adolescents between 3 and 15 years of age. Statistically significant gender differences were found especially with regard to physical activity: boys met the WHO physical activity recommendations slightly more often than girls, and they also used sports activities in school sports activity programmes and sports clubs or studios more often than girls.

In contrast, no statistically significant gender-related differences were found in general health and subjective mental health, which were predominantly rated as good, very good or excellent for both genders. Boys more frequently experienced an increased need for care and support, though this was seen in the youngest age group between 3 and 6 years of age only. Age-related gender differences were also found in the overall physical activity, especially at younger age: Boys between 3 and 10 years of age were more likely to engage in moderate to vigorous physical activity; in the group of older children and adolescents (11–15 years), the difference between boys and girls was no longer statistically significant.

With regard to the questions that explicitly referred to changes during or due to the COVID-19 pandemic, no differences between girls and boys were found in the parent-reported responses. This was true, for example, for deteriorations (or improvements) in subjective mental health during the COVID-19 pandemic. Also, girls and boys were equally affected by cancellations or reduced use of sports activities attributable to the COVID-19 pandemic.

### 4.2 Classification of the results and derivation of recommendations for action

The results of the present study give no indication that the general health of girls and boys between 3 and 15 years of age is assessed differently by the parents. Likewise, in KiGGS Wave 2 (2014–2017), no differences were found regarding the parent-reported general health of girls and boys between 3 and 17 years of age [32]. Gender differences in general health, on the other hand, are more likely to be found in studies on older children and adolescents to the disadvantage of female adolescents [32]. However, a comparison of the results is limited by the different age grouping, since the age group of 16- and 17-year-old adolescents was not included in this evaluation of the KIDA study. The fact that a larger proportion of the male adolescents rate their general health as good to excellent as compared to their female counterparts was also observed, in particular, in studies based on self-reporting by adolescents [40]. Therefore, the absence of a gender difference in the present study may well be due to the fact that the assessments were made exclusively by the parents. For example, [41] showed with regard to health issues that no gender differences were found in the parents’ assessment, while the adolescents themselves – female adolescents in particular – reported significantly more internalising anomalies, such as anxiety or dejection. Both the extent and the existing gender differences were underestimated by the parents [41]. With regard to younger children, though, it is necessary to rely on the parents’ assessment as a proxy. Moreover, the assessment of general health is a valid global indicator that combines physical, mental, and social dimensions of health and well-being into a single item [42, 43]. However, this multidimensionality may mask specific differences in health between girls and boys which thus may not be detectable [44, 45]. In further analyses, more specific indicators for measuring different aspects of health should therefore be used in addition to global overview measures in order to be able to report differences in health by gender in a more differentiated manner.

For subjective mental health, a clear majority of girls and boys are reported by their parents to be in excellent to good mental health. However, according to the parents’ assessment, the mental health of about 20% of the children and adolescents deteriorated during the COVID-19 pandemic as compared to their previous status. Analogous to the findings on general health, no statistically significant differences by gender were found in the KIDA study either in the subjective mental health or in its change. Only a tendency towards ‘fair’ or ‘poor’ mental health was found more frequently for girls between 11 and 15 years of age than for boys of the same age. These gender differences in subjective mental health were also found in a survey from Canada conducted before the pandemic. Here, girls between 12 and 14 years of age were more likely to rate their mental health as ‘fair’ or ‘poor’ than boys of the same age [46]. This is consistent with the fact that upon the onset of adolescence, mental health problems are reported more frequently in girls in the context of increasing internalising problems such as anxiety and depression [44]. During pre-adolescence, though, mental health problems predominate in boys rather than girls of the same age due to a higher prevalence of externalising problems (ibid.). Looking at a deterioration of mental health, further results from Germany showed that during the pandemic, the proportion of girls with a reduced health-related quality of life was higher than of boys [3]. The inconsistent findings so far indicate that further research is needed on gender-related risks for mental health, but also on gender-related resources and coping strategies [47].

Finding that boys between 3 and 6 years of age were more likely than their counterpart girls to have special care and support needs confirms results from the KiGGS study (Basiserhebung (baseline survey), 2003–2006), which found higher prevalences of increased care needs for boys than for girls, with the strongest differences among children between 3 and 10 years of age [37]. Just under 11% of all children and adolescents have longer-term increased care and support needs according to their parents’ responses in KIDA. More research is needed to find out whether the health or social system is able to meet these increased needs, or whether they are unmet (healthcare) needs for which new structures or services need to be established [48]. For this purpose, the existing care and support needs would have to be differentiated in more detail. Initial indications of this come from an interim evaluation of a more in-depth online survey of the KIDA sample from the period of April to August 2022 [49]. It was apparent that this concerned primarily educational and/or mental health services needs rather than medical care needs. It is therefore important to establish gender-sensitive structures through which children and adolescents with poorer general and mental health can be identified at an early stage and referred for help and support, e.g. in educational institutions or in outpatient medical care [4]. Possible gender differences in support needs should be taken into account in facilities for counselling (e.g. family and social counselling centres or school counselling) or support (e.g. family education centres, youth work, outpatient services, preparation for school or maternity centres). Day care centres should also be sensitised to gender-related differences in educational support needs, as differences between boys and girls in KIDA at preschool age were statistically significant. However, some experts also express concerns that ‘typical boy behaviour’ may not infrequently be interpreted as being pathological. If, for example, boys stand out through restlessness and risk-seeking behaviour, this can be part of their normal development, in which they try out behaviour that is perceived as masculine. It does not necessarily have to be considered a disorder on a pathological level that requires increased support [50].

Specific and gender-related support needs are also evident for behavioural risk factors. The analyses with the KIDA data confirm results from earlier national as well as international studies that show that girls are not as physically active as boys [51–53]. This was evident in the KIDA results, in particular in children of nursery or primary school age. A systematic review of the activity of 5- to 17-year-olds in the COVID-19 pandemic confirms that the discrepancies in the physical activity behaviour of boys and girls that were widespread before the pandemic were also observed during the pandemic in the majority of international studies [54]. Various reasons for these gender differences have been described in the literature. During socialisation, for example, different gender-related role models can lead to sports often being seen in a positive context with male identity, and boys place more value on sporting competitions and successes than girls [55, 56]. Negative experiences in physical education are reported more often for girls, which may also have an unfavourable effect on their extracurricular physical activity behaviour [55]. Organised sports, in which a considerable proportion of young people’s physical leisure time activity takes place, also play an important role. The KIDA results show a gender-related difference in their participation in organised sports, which is statistically significant for sports club or commercial offers as well as for participation in voluntary sports activity programmes at school. It is possible that the structure and/or culture of organised sports, especially of the sports clubs, favours boys. This may be a consequence of gender bias in the structure of the offers made by clubs and sports clubs, which, for example, offer more competitive team sports such as football, handball or basketball, which appeal more to boys than girls [55]. Especially in order to compensate for limitations in physical activity such as those brought about by the COVID-19 pandemic, it is important to create offers that are attractive to girls and boys alike. Public health measures should focus specifically on increasing the physical activity of girls. This requires studies that identify specific barriers and promoting factors for the physical activity of girls in everyday life as well as for their participation in organised sports [54]. When organising community sports programmes – whether in clubs or through physical activity programmes at school – care must be taken to respond to the perspectives and interests of girls and boys and to offer a diverse range of sports and courses in order to reduce gender inequalities [55].

### 4.3 Limitations

Due to the sample size, the subgroups were sometimes relatively small in the simultaneous stratification by both gender and age. This led to the results, to some extent, being associated with a relatively large statistical uncertainty, and gender differences may have remained undetected.

The results originate from the telephone survey of the KIDA study. Telephone surveys can be associated with methodological limitations such as selection bias or socially desirable response behaviour [57]. People in the low education group were often less willing to participate in the study, particularly in telephone surveys. Respondents from the low education group are clearly under-represented in the KIDA sample as well. Deviations between the surveyed sample and the population distribution in Germany with regard to age, gender, and region of residence are also possible. In order to address these circumstances and to be able to determine meaningful results, a corresponding design and adjustment weighting was carried out that takes the aforementioned aspects into account [57]. For example, respondents from the low education group were given a high weighting, thus attaching more weight to the answers and doing justice to the lower response rate this group of persons. Furthermore, all interviews in the scope of the KIDA study were conducted in German. Parents with little or no knowledge of German could not, or only to a limited extent, take part in the study.

Another limiting factor concerns the interpretation of comparisons between the current situation and the situation before the onset of the COVID-19 pandemic: The underlying data for the analyses were collected more than two years after the onset of the pandemic. The results therefore only allow limited conclusions to be drawn about whether the health of girls and boys has deteriorated as a result of the pandemic or whether gender differences have changed over time. Over a period of two years, many changes take place in the development of children and adolescents, regardless of external influences such as a pandemic. For this reason, pandemic-related and development-related changes cannot be differentiated unambiguously.

The indicator of subjective mental health was surveyed for the first time for children and adolescents in Germany in the KIDA study, as was the question on total physical activity, which takes into account the new WHO minimum recommendations (an average of one hour of moderate to vigorous exercise per day instead of exactly one hour every day). Therefore, no German reference data are available thus far, which is why a comparison with pre-pandemic time points cannot be made. The same applies to the assessment of mental health in a comparison of during versus before the COVID-19 pandemic, and to the pandemic-related changes in the utilisation of available sports activities.

Various items of physical and mental health were addressed in the telephone survey in KIDA. In order to allow for this broad range of aspects addressed, many aspects could only be queried in abbreviated manner. This also applies to the CSHCN screener, as only one of its 5 items was included in the telephone survey. This allows for an exploratory assessment of the proportion of children with increased care or support needs, but may underestimate the actually existing need. The comparison with previous population-based surveys is therefore also possible to a limited extent only. The survey with the full screening instrument is conducted in a more in-depth online survey of the same respondents, the results of which have not yet been reported here.

### 4.4 Conclusion and outlook

The results of the KIDA survey show that the health of children and adolescents between 3 and 15 years of age is predominantly assessed positively by their parents after approximately three years of the pandemic, whereby statistically significant differences between girls and boys were seen above all in certain age groups and in physical activity. However, gender should not be considered isolated, but always embedded in people’s social environment.

Further analyses should therefore, in the sense of the intersectional approach [58], also take into account other social determinants in addition to gender, such as the socioeconomic situation or migration history. An earlier interim evaluation from the KIDA study [49] shows that girls and boys growing up in families with low educational status and low income suffered the most under the restrictions during the pandemic and were more likely to have developed dysfunctional coping strategies than their age-peers growing up in socially better-off circumstances [59, 60]. Experiences of the loss of a parent’s job or severe family conflicts might also accumulate in different ways with the stresses on girls and boys during the pandemic and thus contribute – possibly only in the longer term – to an increase in differences in the health of socially disadvantaged girls and boys.

Furthermore, it would make sense to survey and analyse the health status of girls and boys using differentiated health indicators, since there are often gender differences in specific health issues [17]. Global health indicators were utilised in the present evaluation.

There is a need for further research with regard to questions like whether the health and health behaviour of girls and boys has changed as a result of the pandemic and, if so, whether gender differences in health have increased or decreased (trend analyses). In order to be able to depict differences in the health of girls and boys over the course of time, long-term surveillance of the health of children and adolescents is required. In addition, the sample must be sufficiently large to be able to carry out differentiated analyses and simultaneously include different age groups, different social determinants, and gender, also in the sense of the intersectional approach. Building on a comprehensive gender-sensitive data collection, corresponding aspects should be integrated into the training and further education of educational, medical, and psychotherapeutic professionals in order to enable boys and girls to grow up in a manner that is fair to both genders.

## Key statement

- There were no gender differences in the subjective general and mental health of children and adolescents between 3 and 15 years of age.
- Only in the age group of 3 to 6 years did boys show an increased need for care or support more often than girls.
- Boys more often met the physical activity recommendations of the World Health Organisation (WHO) than girls, and also utilised sports activities more frequently.
- Gender needs to be studied in the context with other social determinants over an extended period of time to assess the impact of the COVID-19 pandemic in more detail.

## Figures and Tables

**Table 1:** Sample description with unweighted data Source: own diagram

Survey period 09.02.-14.10.2022	Girls n=1,639		Boys n=1,838		Total n=3,478	
Variable					Number (%)	
**Gender of the child**						
Female					1,838	(52.9)
Male					1,639	(47.1)
Diverse					0	(0.0)
No information provided					1	(0.0)
**Age of the child (age group)**						
3–6 years	431	(26.3)	485	(23.4)	917	(26.4)
7–10 years	493	(30.1)	534	(29.1)	1,027	(29.5)
11–15 years	715	(43.6)	819	(44.6)	1,534	(44.1)
**Highest education group of persons in the household (CASMIN)**						
Low education group	42	(2.6)	59	(3.2)	101	(2.9)
Medium education group	539	(32.9)	556	(30.3)	1,095	(31.5)
High education group	1,057	(64.5)	1,223	(66.5)	2,281	(65.6)
Missing values	1	(0.1)	0	0	1	(0.0)
